# A Dynamic Nomogram to Predict the Risk of Stroke in Emergency Department Patients With Acute Dizziness

**DOI:** 10.3389/fneur.2022.839042

**Published:** 2022-02-18

**Authors:** Ying Bi, Fei Cao

**Affiliations:** Department of Neurology, Union Hospital, Tongji Medical College, Huazhong University of Science and Technology, Wuhan, China

**Keywords:** dizziness, stroke, dynamic nomogram, LASSO regression, prediction model

## Abstract

**Objective:**

To develop a risk prediction tool for acute ischemic stroke (AIS) for patients presenting to the emergency department (ED) with acute dizziness/vertigo or imbalance.

**Method:**

A prospective, multicenter cohort study was designed, and adult patients presenting with dizziness/vertigo or imbalance within 14 days were consecutively enrolled from the EDs of 4 tertiary hospitals between August 10, 2020, and June 10, 2021. Stroke was diagnosed by CT or MRI performed within 14 days of symptom onset. Participants were followed-up for 30 days. The least absolute shrinkage and selection operator (LASSO) logistic regression analysis was conducted to extract predictive factors that best identified patients at high risk of stroke to establish a prediction model. Model discrimination and calibration were assessed and its prediction performance was compared with the age, blood pressure, clinical features, duration, and diabetes (ABCD2) score, nystagmus scheme, and finger to nose test.

**Results:**

In this study, 790 out of 2,360 patients were enrolled {median age, 60.0 years [interquartile range (IQR), 51–68 years]; 354 (44.8%) men}, with complete follow-up data available. AIS was identified in 80 patients. An online web service tool (https://neuroby.shinyapps.io/dynnomapp/) was developed for stroke risk prediction, including the variables of sex, trigger, isolated symptom, nausea, history of brief dizziness, high blood pressure, finger to nose test, and tandem gait test. The model exhibited excellent discrimination with an area under the receiver operating characteristic (ROC) curve (AUC) of 0.889 (95% *CI*: 0.855–0.923), compared with the ABCD2 score, nystagmus scheme, and finger to nose test [0.712 (95% *CI*, 0.652–0.771), 0.602 (95% *CI*, 0.556–0.648), and 61.7 (95% *CI*, 0.568–0.666) respectively].

**Conclusion:**

Our new prediction model exhibited good performance and could be useful for stroke identification in patients presenting with dizziness, vertigo, or imbalance. Further externally validation study is needed to increase the strength of our findings.

## Introduction

It has been established that stroke accounts for 2–13.4% of patients presenting to the emergency department (ED) with dizziness (1–5). Stroke is the second leading cause of death globally and has a limited treatment time window (6–8). Misdiagnosis can affect treatment decision-making, thus seriously impacting disease outcomes and patient quality of life, emphasizing the importance of timely stroke diagnosis in patients with dizziness (9–11). In this respect, it has been shown that over one-third of stroke cases are missed at the first visit by emergency physicians (EPs) (12). The misdiagnosis rate can be as high as 24–60%, especially when the symptoms are mild, non-specific, and transient (13).

Many efforts have been made to differentiate stroke from other causes of dizziness. The age, blood pressure, clinical features, duration, and diabetes (ABCD2) score with an area under the receiver operating characteristic (ROC) curve (AUC) of 0.79, and head impulse, nystagmus pattern, test of skew (HINTS) test with high sensitivity/specificity (100%/96%), are the two most widely acknowledged tools for stroke identification in patients with acute dizziness (14–17). The features of posterior circulation stroke, which had a triple misdiagnosis rate than that of anterior circulation stroke, are not included in ABCD2 score, which could probably decrease its diagnostic accuracy (18–21). Recently, a combination of ABCD2 ≥ 4 and a central pattern of nystagmus has been shown to yield higher sensitivity than the ABCD2 score alone for identifying stroke (22). Notwithstanding that the HINTS is reported to yield good diagnostic performance, studies report their low usage during clinical practice in EDs, as only 30% of EPs agree with the use of HINTS in patients with dizziness, not to mention the usage of head impulse, nystagmus pattern, test of skew, acute hearing loss (HINTS-plus) (23). Truncal ataxia, an easy-to-evaluate test, has been shown to yield high sensitivity to differentiate stroke from acute vestibular syndrome when combined with the nystagmus test (24). Posterior circulation ischemia (PCI) score, TriAGe+ score, STANDING algorithm, and DEFENSIVE scale were recently studied to estimate the risk of stroke in patients with dizziness (25–28). NLR, S100B, and NSE have been reported as new blood markers for the prediction of stroke in patients with dizziness (29–31). However, most of these prediction approaches were developed with small sample sizes, retrospectively collection, no validation, and have not been applied in clinical practice. Early risk stratification of dizzy patients is crucial, and misdiagnosis of stroke can lead to serious complications and poor outcomes. Thus, it is still challenging to discriminate stroke from patients with dizziness in the ED.

The present study sought to develop and validate a clinical prediction model based on easy-to-get predictors to identify patients with dizziness at risk of stroke in the ED.

## Methods

### Study Design and Setting

This was a multi-center, observational study that prospectively enrolled patients from August 10, 2020, to June 10, 2021. The study was conducted in the EDs at Wuhan Union Hospital, Wuhan Union Hospital West Campus, Wuhan Fourth Hospital (Puai Hospital), and Hubei Provincial Hospital of Integrated Chinese and Western Medicine. All study centers were teaching hospitals and home to the standardized resident training program. The four hospitals have a combined annual ED visit of ~200,000 patients, providing 24-h neurological consultation and emergency neuroimaging facilities.

The study was approved by the Ethics Committees of Tongji Medical College, Huazhong University of Science and Technology (S227) and was registered in the Chinese Clinical Trial Registry (www.chictr.org.cn, ChiCTR2000037496). The research was conducted in accordance with the Declaration of Helsinki and written informed consent was obtained from all participants.

### Participants

Adult patients presenting with dizziness/vertigo or imbalance at the EDs were consecutively screened by 2 trained investigators *via* the ED triage electronic medical record system 24 h daily. Dizziness/vertigo and imbalance were defined in accordance with the consensus document of Bárány Society (32). Patients were excluded for (1) more than 14 days duration since symptom onset at admission, (2) chronic recurrent symptoms (defined as no less than 5 prior episodes similar in quality, intensity, and duration to the current symptoms, with at least one episode over a year and one within the last year), (3) history of multiple sclerosis, (4) dizziness thought to be the result of trauma, orthostatic hypotension, medication intoxication, hypoglycemia, or a known medical or neurologic disorder (e.g., hepatic encephalopathy and hydrocephalus), (5) central nervous system (CNS) examination abnormalities (e.g., hemiparesis, hemisensory loss, and gaze palsy) with a National Institutes of Health Stroke Scale (NIHSS) ≥5 [mild abnormalities (e.g., mild axial ataxia, dysarthria, or sensory symptoms) were not excluded], (6) patient refusal of neuroimaging tests or with contraindications to MRI or CT, or (7) refusal to participate.

The data of eligible patients were collected through detailed interviews by 2 investigators using a structured case report form customized for this study and blinded to neurologic exam abnormalities and primary outcome measures, such as CT and MRI.

### Baseline Clinical Measurements

Baseline clinical variables, such as demographic characteristics, presenting symptoms, and medical history were obtained directly from the participants. A standardized physical examination was performed by two neurologists well trained in neuro-otology before the CT and/or MRI exam or blinded to the neuroimaging results. The physical examination included ocular motor examination, visual field assessment, assessment of cranial nerves, muscle strength, sensation, coordination, balance, Dix-Hallpike test, and hearing impairment by finger rub. During the nystagmus test, the eyes of patients were observed by investigators in primary position (straight-ahead) and then sequentially with gaze directed ~30–40 degrees left, right, up, and down. In each position, the vector of the fast-beating component and the intensity of nystagmus were recorded. In addition, the nystagmus examinations were recorded by video and estimated by a third investigator. A positive result, central pattern of nystagmus, was identified when any of bidirectional gaze-evoked nystagmus (GEN), persistent vertical nystagmus scored in any position, or isolated torsional nystagmus was observed (16). Bidirectional gaze-evoked nystagmus was defined as the presence of right-beat nystagmus when looking to the right and left-beat nystagmus when looking to the left, with the nystagmus lasted longer than 5 s. Any other nystagmus or no nystagmus were recognized as negative. During the finger to nose test, participants were asked to touch the tip of their nose with the index finger and repeatedly move the index finger to touch the finger of examiner and back to the nose (33). A positive result was determined if the subject tended to push the test finger upward, toward the ceiling and above the line between the finger of examiner and the nose of subject, showing dysmetria (34). A tandem gait test was conducted, and a positive test was observed when the patient could not walk 10 consecutive steps straight in the heel-to-toe manner with eyes open in up to 2 attempts (35–37). The ABCD2 score, NIHSS, modified Rankin Scale (mRS), and the Barthel index were calculated at the first visit.

From the electronic medical record system, we collected data on the arrival time, vital signs, such as body temperature, heart rate, and blood pressure, first laboratory tests of blood routine, kidney function, and coagulation parameters within 24 h after symptoms onset, and other routine laboratory parameters and administered medications by two investigators independently. Investigators were blinded to the neuroimaging findings and neurological exam abnormalities when they extracted the data.

### Outcome Measures

The outcome was an imaging-based definition of AIS according to the international diagnostic guidelines. All enrolled patients completed either CT or MRI with diffusion-weighted imaging (DWI) and apparent diffusion coefficient of the brain within 14 days after symptoms onset. Besides, the MRI was performed more than 48 h from symptom onset to reduce the missed diagnosis due to false-negative findings (38, 39). Diffusion-weighted MRIs were performed with either a 1.5 or 3 T MRI machine and the slice thickness was 5 mm with a 1.5 mm slice gap. The original images from the 4 research centers were interpreted by two board-certified neuroradiologists blinded to the clinical information.

To rule out the possibility of missed stroke diagnosis and avoid verification bias, all patients enrolled were followed-up by a structured telephone interview a month after onset. Participants were asked whether they had undergone any new neuroimaging test after discharge and were requested to provide imaging results once they had. Moreover, we checked for revisits to the ED or hospitalization using the electronic medical record system.

### Predictor Variables

We searched for predictors of stroke from keynote papers (3, 31, 40–50). Candidate predictors were identified when the factors were consistently reported in predictive research on patients with dizziness, easily ascertained, and routine diagnostic tests for patients with stroke. The candidate predictors employed for the development of a novel risk prediction model in our study are listed in Table 1. Dizziness/vertigo or imbalance with “trigger” was defined as symptoms presenting with an obvious trigger. Under most circumstances, a reproducible and repetitive relationship between the trigger stimulus and dizziness spell should be present. “Isolated symptom” was defined as dizziness/vertigo or imbalance without any other neurologic findings. “History of brief dizziness” was defined as a brief episode of dizziness that occurred within 3 months before the attack except for chronic recurrent symptoms. “Sleep disorder” was defined as sleep difficulty symptoms reported by the patient himself. High blood pressure was defined as systolic blood pressure (SBP) ≥ 140 mm Hg or diastolic blood pressure (DBP) ≥ 90 mm Hg after arriving at the ED.

**Table 1:** Candidate predictors in this study.

	**Candidate predictors**
General information of patients	Age, sex
Presenting symptoms	Duration, trigger, isolated symptom, nausea, headache
Medical history	Hypertension, diabetes mellitus, atrial fibrillation, cardiovascular disease, stroke history, history of brief dizziness (within 3 months), sleep disorder, family history of stroke
Other risk factor	Current smoke, current drinking
Physical examination	High blood pressure, finger to nose test, tandem gait test

### Statistical Analysis

Data analysis was conducted using R (version 4.0.4; www.r-project.org) and SPSS (version 23.0; IBM, Chicago, IL, USA). Multiple imputation was used to deal with missing data. Values of missing variates (NLR, Hct, hsCRP, and uric acid) were imputed by assuming that data were missing at random with 50 imputations. All candidate predictor variables and several relevant variables were included in the imputation model. Predictive mean matching and logistic regression were applied to impute continuous and binary variables, respectively. The normality test was performed using the Shapiro–Wilk test. Continuous variables with a normal distribution were reported as mean ± SD, and those with a non-normal distribution were reported as median (interquartile range, IQR). Categorical variables were presented as numbers and percentages. The *t*-test or Mann–Whitney *U*-test was conducted to compare continuous variables. Comparisons of categorical variables were performed using Fisher's exact test.

#### Model Development

For the model development, the least absolute shrinkage and selection operator (LASSO) logistic regression was conducted to select variables that best identified patients at high risk of stroke through the selection of tuning parameter lambda (λ) (51, 52). The LASSO penalization factor selected predictors by shrinking coefficients for weaker predictors toward zero. The variables with a non-zero coefficient were chosen as stroke risk predictors. To avoid overfitting models, a 10-fold cross-validation was used for λ selection. Then, the multivariate logistic regression was used to compute the adjusted odds ratios (*OR*s) of the chosen predictors.

A nomogram was built to provide visualized risk prediction based on the multivariable analysis results using the “rms” R package. In the nomogram, the predicted probabilities were mapped into points on a scale from 0 to 100 in a user-friendly way. The point system works by ranking the effect estimates, regardless of the statistical significance, and it is influenced by the presence of other covariates in this model. The effect with the highest β (absolute value) will be assigned 100 points on the scale, and the remaining variables are assigned a smaller number of points proportional to their effect size (53). The total points accumulated by all the selected variables correspond to the predicted probability for a patient (54). Therefore, nomograms provide personalized predictions based on the specific clinical parameters of patient through an easy-to-use manner. Finally, to facilitate individualized prediction in clinical practice, we constructed a web-based calculator, written in R code using the Shiny framework (http://www.shinyapps.io/).

#### Model Evaluation

To evaluate the stability of the model and to perform model validation, a bootstrap resampling approach was used with 1,000 iterations. Calibration of the models was evaluated using Hosmer–Lemeshow goodness-of-fit test and a calibration curve was plotted to compare the agreement between the actual stroke rates and the model predicted stroke probabilities. A 45 degrees diagonal reference line denoted the line of perfect calibration, whereas deviation above or below this line reflects underprediction or overprediction. The discrimination power of the model was assessed through an ROC curve. The AUC and Harrell's concordance index (C-index) were measured to assess the prediction performance of the model. AUCs for the prediction model and other existing methods of risk prediction were compared using the DeLong's test. To determine the clinical utility of the model, decision curve analysis (DCA) was performed to quantify the net benefits at different threshold probabilities. This method is based on the principle that the relative harm of false positives and false negatives can be represented by probability threshold. The net benefit is obtained by subtracting the proportion of patients showing false positive results from the proportion showing true positive results, and then weighing the relative harm of false positive and false negative results (55). A good model usually yields a high net benefit over a wide range of threshold probabilities in the DCA curve. The following R packages were mainly used for our analysis: “glmnet” for LASSO Logistic regression algorithm; “pROC” for ROC analyses and DeLong's test; “rms” for calibration curve; and “rmda” for decision curve analysis. A two-sided *p* < 0.05 was considered statistically significant.

The present study was conducted following the Transparent Reporting of a multivariable prediction model for Individual Prognosis or Diagnosis (TRIPOD) guidelines (56).

## Results

### Patient Characteristics

**Figure 1:**
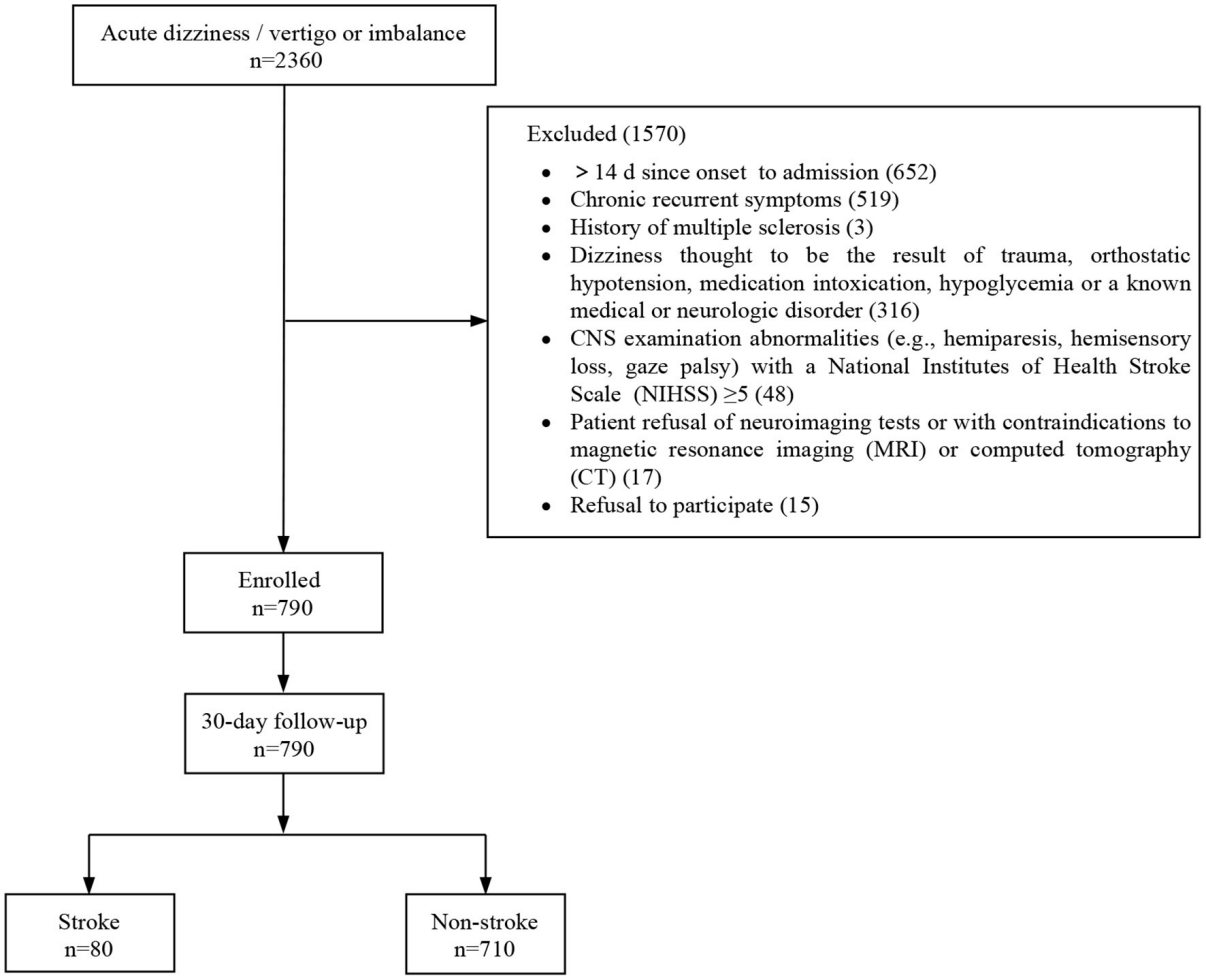
Flowchart of patient enrollment.

In this study, 790 out of 2,360 patients who visited the EDs with dizziness/vertigo or imbalance were finally included. A total of 1,570 patients were excluded for the following reasons: time from symptom onset to admission longer than 14 days (*n* = 652), chronic recurrent symptoms (*n* = 519), history of multiple sclerosis (*n* = 3), patients with a history of trauma, orthostatic hypotension, medication intoxication, hypoglycemia, or a known medical or neurologic disorder (*n* = 316), CNS abnormalities with an NIHSS higher than 5 (*n* = 48), patient refusal or contraindicated for neuroimaging test (*n* = 17), and refusal to participate (*n* = 15) (Figure 1). All patients who met the inclusion criteria underwent neuroimaging; 628 (79.5%) underwent CT, and 456 (57.7%) underwent MRI. Data for 30-day follow-ups were available for all participants. Baseline clinical characteristics of the study population are presented in Table 2. A stroke was diagnosed in 80 patients (10.1%), and 59 (73.8%) were related to posterior circulation ischemia.

**Table 2:** Demographic and clinical characteristics of 790 patients with dizziness/vertigo or imbalance.

**Characteristic**	**Total (*n* = 790)**	**Patients without stroke (*n* = 710)**	**Patients with stroke (*n* = 80)**	***P*-value**
Age (median [IQR])	60.00 [51.00, 68.00]	60.00 [51.00, 68.00]	62.50 [53.00, 69.00]	0.171
Male	354 (44.8)	289 (40.7)	65 (81.2)	<0.001
BMI (median [IQR])	23.88 [21.97, 25.95]	23.88 [21.88, 25.94]	24.90 [22.73, 26.45]	0.03
Type				
Vertigo	413 (52.3)	376 (53.0)	37 (46.2)	0.108
Dizziness	286 (36.2)	258 (36.3)	28 (35.0)	
Imbalance	91 (11.5)	76 (10.7)	15 (18.8)	
Persistent or transient				<0.001
Persistent	254 (32.2)	213 (30.0)	41 (51.2)	
Transient	536 (67.8)	497 (70.0)	39 (48.8)	
Duration				0.012
<1 min	153 (19.4)	145 (20.4)	8 (10.0)	
1–9 min	153 (19.4)	139 (19.6)	14 (17.5)	
10–60 min	130 (16.4)	121 (17.0)	9 (11.2)	
>60 min	354 (44.8)	305 (43.0)	49 (61.3)	
Classification				<0.001
Episodic vestibular syndrome	531 (67.2)	492 (69.3)	39 (48.8)	
Acute vestibular syndrome	259 (32.8)	218 (30.7)	41 (51.2)	
Trigger	388 (49.1)	366 (51.5)	22 (27.5)	<0.001
Arrival by emergency ambulance	169 (21.4)	140 (19.7)	29 (36.2)	0.001
Isolated symptom	613 (77.6)	575 (81.0)	38 (47.5)	<0.001
Time, onset to admission, h (median [IQR])	12.00 [3.00, 72.00]	11.00 [3.00, 72.00]	19.00 [4.00, 48.00]	0.396
Time, onset to last CT, h (median [IQR])	9.00 [3.11, 37.98]	9.00 [3.00, 35.72]	8.93 [3.68, 46.57]	0.442
Time, onset to MRI, h (median [IQR])	112.01 [72.00, 169.25]	114.00 [73.55, 171.85]	98.50 [66.00, 152.00]	0.216
**Vital sign, median (IQR)**				
HR (median [IQR])	76.00 [68.00, 83.00]	76.00 [69.00, 81.00]	77.50 [67.00, 86.25]	0.425
SBP (median [IQR])	141.00 [128.00, 157.00]	140.00 [127.00, 155.00]	155.50 [141.00, 168.50]	<0.001
DBP (median [IQR])	86.00 [79.00, 95.00]	86.00 [78.00, 95.00]	91.50 [85.00, 101.00]	<0.001
High blood pressure ^a^	495 (62.7)	427 (60.1)	68 (85.0)	<0.001
**Medical history**				
Hypertension	393 (49.7)	339 (47.7)	54 (67.5)	0.001
Diabetes mellitus	148 (18.7)	126 (17.7)	22 (27.5)	0.048
Stroke history	158 (20.0)	137 (19.3)	21 (26.2)	0.142
Migraine	175 (22.2)	165 (23.2)	10 (12.5)	0.032
Hyperlipidemia	314 (39.7)	283 (39.9)	31 (38.8)	0.904
History of brief dizziness (within 3 months)	302 (38.2)	282 (39.7)	20 (25.0)	0.011
Sleep disorder	349 (44.2)	327 (46.1)	22 (27.5)	0.002
Family history of stroke	249 (31.5)	211 (29.7)	38 (47.5)	0.002
Current smoke	170 (21.5)	136 (19.2)	34 (42.5)	<0.001
Current drinking	114 (14.4)	95 (13.4)	19 (23.8)	0.018
**Associated symptoms**				
Headache (%)	133 (16.8)	118 (16.6)	15 (18.8)	0.637
Nausea (%)	555 (70.3)	510 (71.8)	45 (56.2)	0.006
Vomit (%)	401 (50.8)	358 (50.4)	43 (53.8)	0.637
Fear of sound (%)	154 (19.5)	137 (19.3)	17 (21.2)	0.657
Phonophobia (%)	162 (20.5)	145 (20.4)	17 (21.2)	0.884
Speechless (%)	54 (6.8)	34 (4.8)	20 (25.0)	<0.001
Hearing loss (%)	59 (7.5)	52 (7.3)	7 (8.8)	0.652
Tinnitus (%)	192 (24.3)	174 (24.5)	18 (22.5)	0.784
Facial paresthesia (%)	34 (4.3)	24 (3.4)	10 (12.5)	0.001
Nervous (%)	129 (16.3)	127 (17.9)	2 (2.5)	<0.001
Difficulty breathing (%)	52 (6.6)	51 (7.2)	1 (1.2)	0.052
mRS (median [IQR])	1.00 [0.00, 2.00]	1.00 [0.00, 2.00]	2.00 [1.00, 3.00]	<0.001
NIHSS (median [IQR])	0.00 [0.00, 0.00]	0.00 [0.00, 0.00]	1.00 [0.00, 2.00]	<0.001
Barthel Index (median [IQR])	90.00 [75.00, 100.00]	90.00 [80.00, 100.00]	72.50 [60.00, 90.00]	<0.001
ABCD2 score (median [IQR])	2.00 [1.00, 4.00]	2.00 [1.00, 3.00]	3.50 [2.00, 5.00]	<0.001
**Examination**				
Ophthalmoplegia (%)	14 (1.8)	8 (1.1)	6 (7.5)	0.001
Facial paralysis (%)	26 (3.3)	11 (1.5)	15 (18.8)	<0.001
Hoarse voice (%)	20 (2.5)	6 (0.8)	14 (17.5)	<0.001
Diplopia (%)	28 (3.5)	17 (2.4)	11 (13.8)	<0.001
Limb weakness (%)	31 (3.9)	18 (2.5)	13 (16.2)	<0.001
Sensory deficit (%)	41 (5.2)	30 (4.2)	11 (13.8)	0.001
Positive finger to nose test (%)	41 (5.2)	20 (2.8)	21 (26.2)	<0.001
Positive tandem gait test (%)	320 (40.5)	268 (37.7)	52 (65.0)	<0.001
Central nystagmus (%)	33 (4.2)	15 (2.1)	18 (22.5)	<0.001
Positive Romberg test (%)	299 (37.9)	254 (35.8)	45 (56.2)	<0.001
**Laboratory test (median [IQR])**				
Time, onset to blood routine (median [IQR])	4.65 [2.50, 10.00]	4.43 [2.50, 9.32]	6.39 [3.79, 17.61]	0.029
NLR	4.56 [2.67, 8.50]	4.55 [2.63, 8.13]	4.90 [3.18, 9.32]	0.465
Hemoglobin (g/dL)	13.80 [12.70, 14.90]	13.60 [12.60, 14.70]	14.60 [13.30, 15.63]	<0.001
Hct (%)	42.10 [38.80, 45.00]	41.80 [38.60, 44.80]	43.75 [41.40, 47.90]	0.001
hsCRP (mg/L)	1.52 [0.71, 3.24]	1.46 [0.65, 2.79]	2.71 [1.27, 5.15]	0.012
BUN (mg/dL)	5.00 [4.08, 6.00]	4.90 [4.01, 6.00]	5.50 [4.33, 6.43]	0.049
Creatinine (μmol/L)	67.50 [59.40, 77.30]	66.55 [58.45, 76.00]	75.40 [67.00, 85.55]	<0.001
Uric acid (μmol/L)	313.30 [257.75, 378.45]	308.00 [256.90, 378.00]	338.50 [272.28, 381.80]	0.127
Time, onset to coagulation test (median [IQR])	4.50 [2.52, 10.28]	4.30 [2.50, 9.35]	6.78 [4.00, 18.00]	0.009
Dimmer (mg/L)	0.29 [0.22, 0.46]	0.28 [0.22, 0.43]	0.36 [0.26, 0.54]	0.031
FIB (g/L)	3.17 [2.69, 3.70]	3.11 [2.67, 3.67]	3.32 [2.96, 3.81]	0.11
HDL (mmol/L)	1.12 [0.94, 1.35]	1.14 [0.96, 1.37]	1.01 [0.86, 1.17]	<0.001
LDL (mmol/L)	2.57 [2.03, 3.20]	2.58 [2.07, 3.21]	2.51 [2.03, 3.08]	0.478
TC (mmol/L)	4.35 [3.74, 5.15]	4.37 [3.77, 5.17]	4.28 [3.46, 4.84]	0.099

*Data are presented as n (%) or median with interquartile range (IQR); a: systolic blood pressure (SBP) ≥ 140 mm Hg or diastolic blood pressure (DBP) ≥ 90 mm Hg*.

### Prediction Model Building

**Figure 2:**
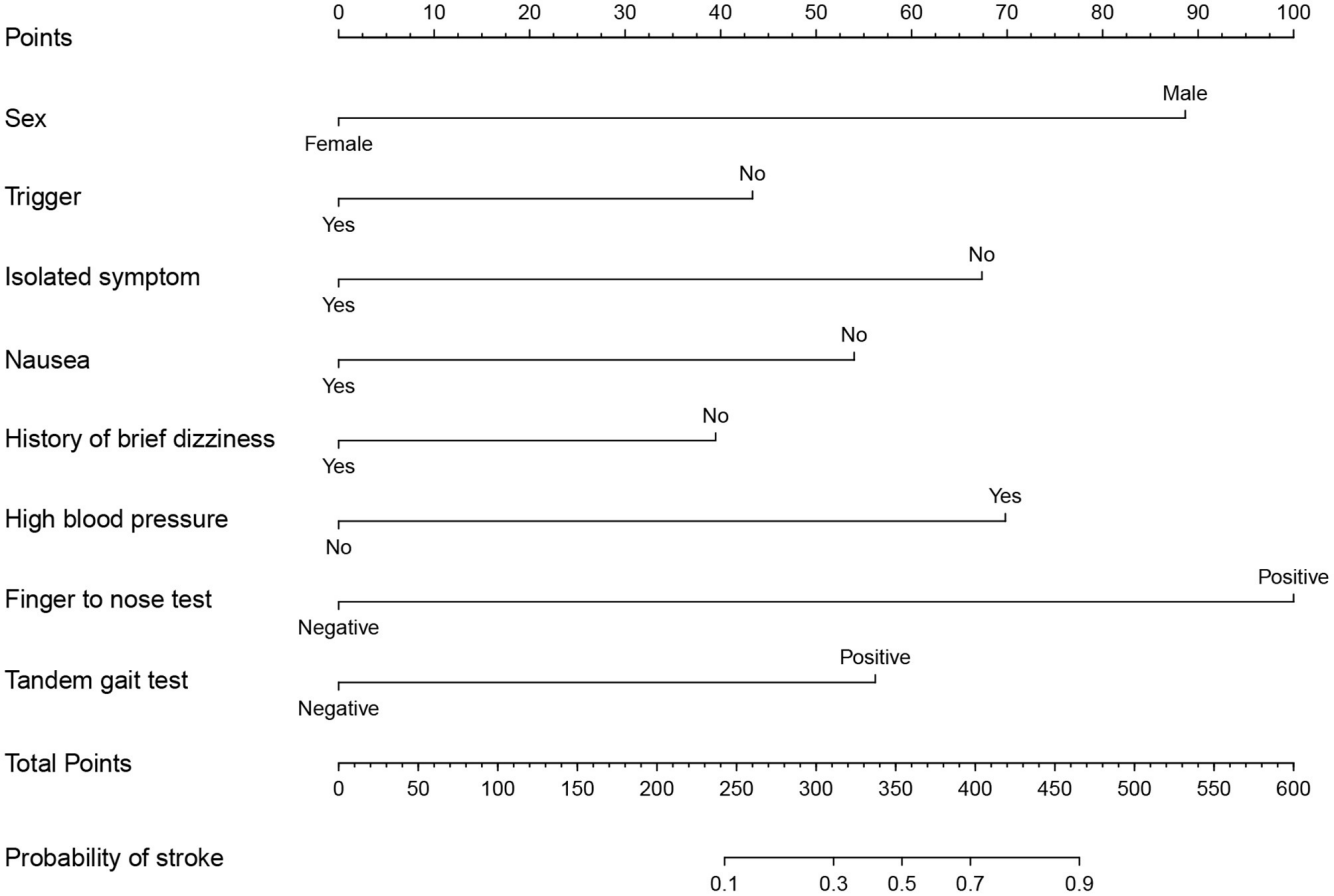
Nomogram to determine the probability of stroke in dizziness/vertigo or imbalance. To use the nomogram, users should locate the sex of a patient on the Sex axis first, and then draw a line straight upward to the Points axis to determine the number of points received for his sex. Repeat this process for all axes of the variables, sum the points achieved for each predictor, and located this sum on Total Points axis. Finally, draw a line straight down from the Total Points axis to determine the Probability of stroke at the lower line of the nomogram.

The results of LASSO analysis and cross-validation illustrated that the optimal value of λ was 0.0201005, with log (λ) = −3.907011, by applying the 1 SE of the minimum criteria (the 1-se criteria) (Figure S1). The multivariate Logistic regression analysis was then performed to establish a prediction model (Table S1). According to the results, 8 out of 20 potential predictors were screened out with non-zero coefficients for model construction. The variables, including sex, trigger, isolated symptom, nausea, history of brief dizziness, high blood pressure, finger to nose test, and tandem gait test, were then incorporated into a nomogram (Figure 2). To use the nomogram, the value of an individual participant is located on each variable axis, and a line is drawn straight upward to the Points axis to determine the number of points received for each variable value, add the points from all the variables. Finally, draw a line straight down from the Total Points axis to determine the Probability of stroke at the lower line of the nomogram. For convenient use during the clinical practice, we developed a web-based calculator (https://neuroby.shinyapps.io/dynnomapp/) based on the nomogram (Figure S2).

### Prediction Model Performance Assessment

**Figure 3:**
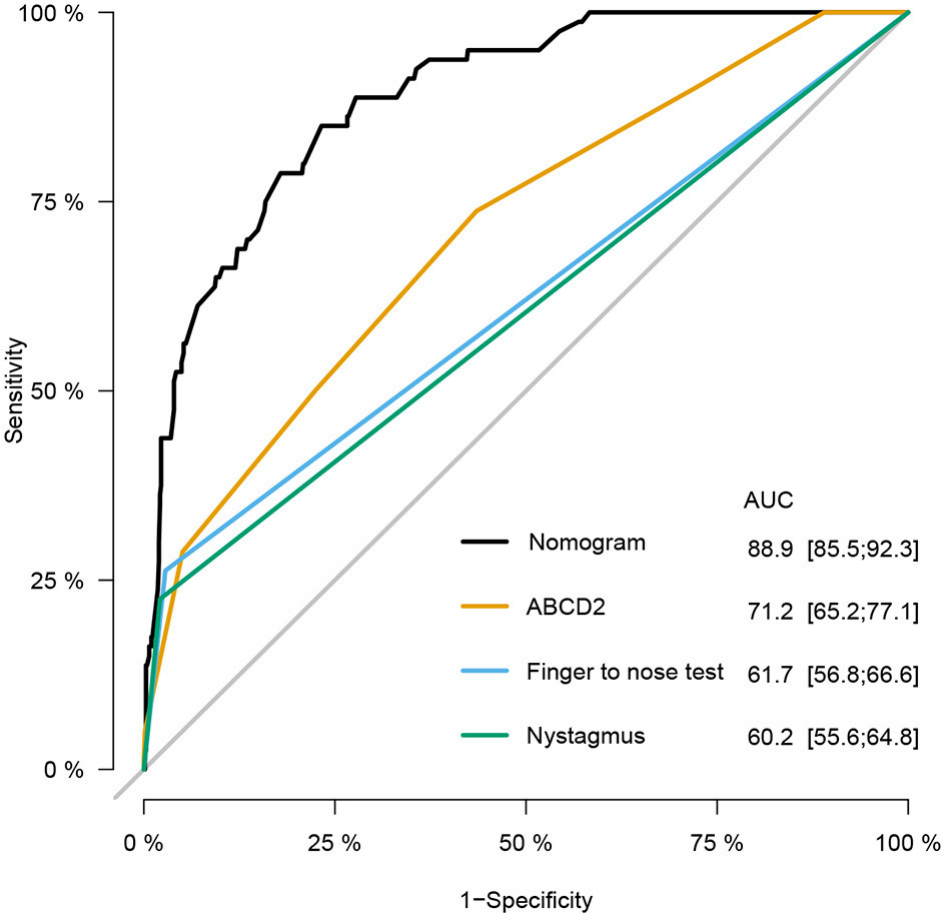
Receiver operating characteristic (ROC) curves of the nomogram, age, blood pressure, clinical features, duration, and diabetes (ABCD2) score, finger to nose test, and nystagmus scheme for stroke prediction at emergency department (ED). Compared with the ABCD2 score, finger to nose test alone, and nystagmus scheme, the nomogram prediction model shows excellent diagnostic efficacy with especially high area under the ROC curve (AUC).

The prediction accuracy of the nomogram was estimated using the Hosmer–Lemeshow goodness-of-fit test yielding a *p* of 0.769, suggesting good prediction accuracy. The calibration plot illustrated in Figure S3 revealed a good consistency between the predicted probability and observed frequency of stroke. To assess the ability of model to discriminate patients with different diagnoses, we calculated the C-statistic. The unadjusted C-statistic of the model was 0.889, and the bootstrap-corrected C index was 0.876, demonstrating excellent discrimination. To compare the predicted value of our model with other established clinical methods, the ROC curves and AUCs of the ABCD2 score, finger to nose test, and nystagmus scheme for stroke prediction in patients with dizziness/vertigo or imbalance were plotted as shown in Figure 3, including an ROC curve of the newly-established model. The results suggested that our nomogram exhibited superior predictive performance with an AUC (95% *CI*) of 0.889 (95% *CI*: 0.855–0.923). The finger to nose test, which was a component of the nomogram, yielded a lower AUC value (0.617, 95% *CI*: 0.568–0.666) than our nomogram. The AUC values of the ABCD2 score and nystagmus scheme were 0.712 (95% *CI*: 0.652–0.771) and 0.602 (95% *CI*: 0.556–0.648).

### Clinical Utility

**Figure 4:**
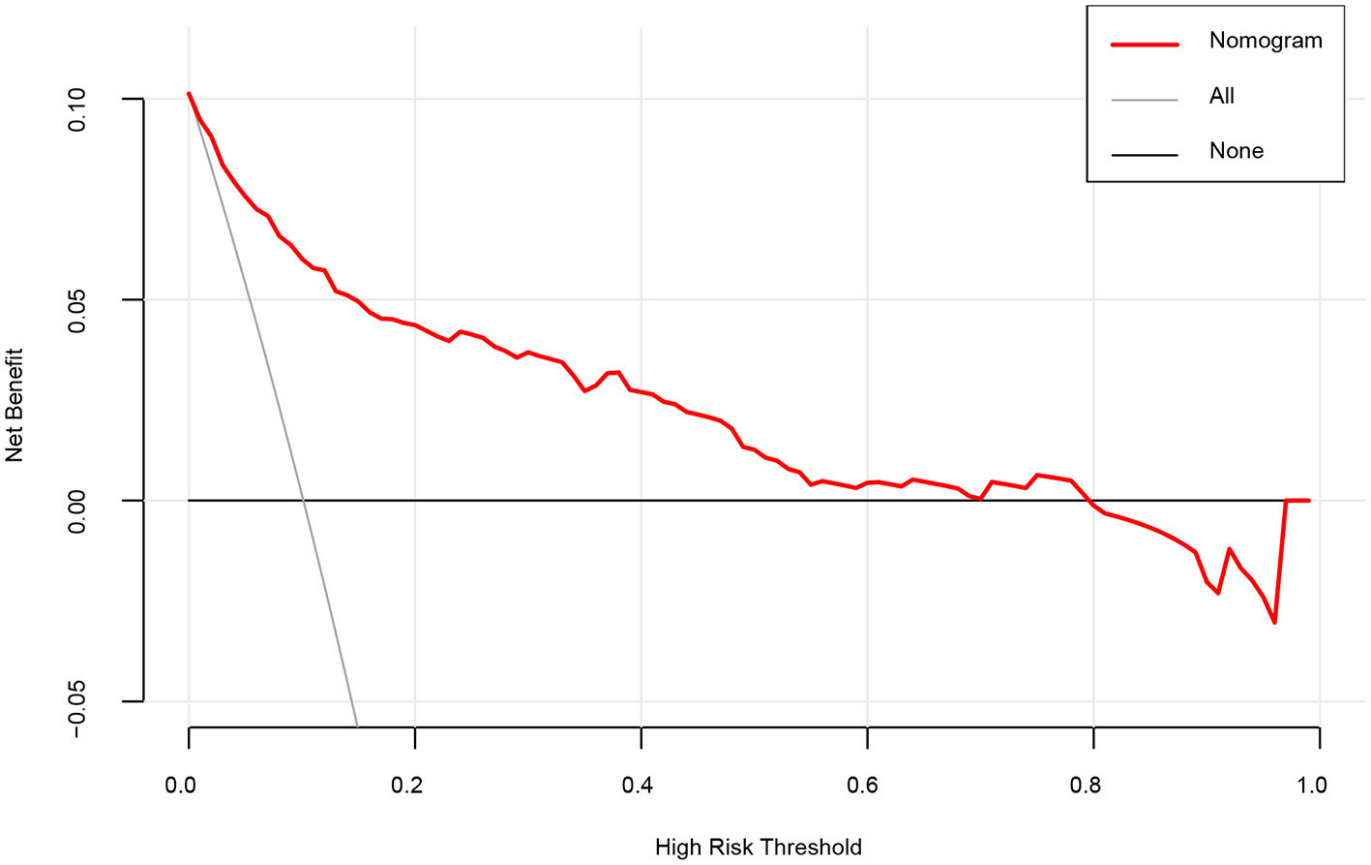
A decision curve analysis (DCA) for the nomogram model to predict stroke. The *x*-axis indicates the threshold probability and the *y*-axis indicates the net benefit. The thick black line represents the net benefit when assume that no patient suffering stroke. The thin gray line shows the net benefit when assume that all patients suffering stroke. As shown by the red curve in the figure, when the threshold probability is <0.79, using our nomogram prediction model yields significant net clinical benefits to patients.

The DCA was applied to evaluate the clinical usefulness of the model. As illustrated in Figure 4, patients with dizziness/vertigo or imbalance would gain clinical benefit from our model if the risk threshold is <79%. For example, if the high-risk threshold probability of a patient is 30%, then the net benefit is 0.364 when we use the model to predict stroke risk.

## Discussion

We developed a novel practical model to predict the risk of stroke in patients presented to the ED with dizziness/vertigo or imbalance. The model was internally validated and exhibited good discrimination and calibration power. The model incorporated eight components: sex, trigger, isolated symptom, nausea, history of brief dizziness, high blood pressure, finger to nose test, and tandem gait test. In addition, we developed a web-based calculator for efficient and individualized prediction, which could assist physicians during clinical decision-making. To the best of our knowledge, this is the first study to construct a nomogram and web-based calculator for predicting the stroke risk of individual patients with dizziness.

Although many studies have been sought to explore novel approaches for early stroke recognition in patients with dizziness, few have been translated into the clinical practice. During the clinical practice, it has been established that misdiagnosis rates are high leading to treatment delay, especially in patients with posterior circulation stroke who exhibit non-specific symptoms and rarely present with unilateral weakness (57–59). It has been reported that 30% of unidentified stroke patients exhibited limb ataxia in EDs (60). Compared with the ABCD2 score, our nomogram model yielded a better diagnostic performance with higher AUC value. We hypothesize that the inclusion of the finger to nose and tandem gait tests might partly account for high accuracy of our model to a certain extent since both are reportedly powerful predictors for posterior circulation ischemia and minor stroke (35, 40, 41). In comparison, ABCD2 score was initially generated to assess the risk of early stroke after a transient ischemic attack and incorporate signs of anterior circulation abnormal and features of medical history (61). As for the HINTS test, the complex oculomotor examination might be the main reason limiting its use (13, 62). In addition, it is hard for patients to corporate to complete the examination, especially when they have persistent vertigo with serious concomitant symptoms, such as nausea, vomit, and postural instability. Unlike the HINTS and the nystagmus scheme, which are challenging for EPs to distinguish, the two tests included in our nomogram were common physical examinations that are easy to implement in the ED (62, 63). Besides, the HINTS test and nystagmus scheme were originally developed and used in patients with acute vestibular syndrome, however, they are not applicable in all patients with dizziness, limiting their clinical utility (64). Besides, skew deviation and head-impulse tests are the key findings for the HINTs. The nystagmus scheme alone will decrease its discrimination of risk prediction. Indeed, the proportion of patients showing pure vertical, torsional, GEN is rather low. These findings may explain the low AUC value associated with the nystagmus scheme in our study. The inclusion and exclusion criteria of the present research determined that the prediction model would have a wide application for patients with dizziness/vertigo or imbalance in clinical practice in the ED.

The acronym TiTrATE (timing, triggers, and targeted bedside eye examinations) is useful for targeting the examinations to help physicians make a specific diagnosis for patients with dizziness (65). The STANDING algorithm is a structured diagnostic algorithm that combined the presence and type of nystagmus, nystagmus direction, head impulse test, and evaluation of the standing position and gait into a four-step sequence (26). However, they are more suitable for use in dizziness clinics rather than EDs which need rapid assessment and triage treatment. The TriAGe+ Score and PCI score are two risk scores for stroke diagnosis in patients with dizziness, of which some components are the same as the variables in our prediction model. The TriAGe+ Score is composed of 8 variables, including triggers, atrial fibrillation, male gender, blood pressure ≥ 140/90 mm Hg, brainstem or cerebellar dysfunction, focal weakness or speech impairment, dizziness, and no history of vertigo or dizziness or labyrinth or vestibular disease, which were derived from a multivariate logistic regression analysis (27). A high sensitivity (96.6%) with a good negative likelihood ratio (0.15) was identified when the cutoff value was determined as 5 points, outperformed the ABCD2 score for stroke prediction. The reason for the failure of the score to be applied clinically may be due to the retrospectively data collection, single center study, and no model validation. The PCI score was established with a high AUC of 0.82 whereas the study patients were retrieved from the department of neurology retrospectively (25). It is not well suited for patients in ED. In comparison, our prediction model, prospectively collected dizziness/vertigo or imbalance patients from 4 EDs, shows high discrimination performance and good prediction accuracy, will be a useful tool for Eps.

The nomogram prediction model incorporated eight variables. Consisting with the literature, the variables of “isolated symptom” and “history of brief dizziness” were inversely associated with stroke onset while “male” and “high blood pressure” on admission were associated with a high risk of stroke in our study (44, 45, 66, 67). “Nausea” and “tandem gait test” were selected as predictors in a stroke risk stratification method for the first time. The “finger to nose test” is an easy-to-perform test for EP and has been proved to be a good predictor of stroke. In fact, addition of the finger to nose test to the emergency medical services was shown to improve stroke recognition (40). Above all, variables incorporated into the nomogram are readily accessible and do not require laboratory tests or instrumental examinations, making the nomogram a simple-to-use prediction model. Furthermore, our developed online web service prediction model can be a powerful and convenient tool for EPs to evaluate the stroke risk in patients with dizziness/ vertigo or imbalance.

## Strength and Limitation

The main strength of our study is that this study was a prospective multicenter observational study that enrolled participants from four different tertiary hospitals. Moreover, predictors incorporated in our nomogram are well defined and readily available. Then, we incorporated the prediction model into an online risk assessment calculator that could be convenient for clinical practice. Besides, our nomogram yielded significantly higher AUC values than previous models reported in the literature.

Nonetheless, the present study has some limitations. First of all, although our model was internally validated by bootstrap resampling to demonstrate its applicability, external validation is essential to increase the robustness of our results. Furthermore, not all participants underwent DWI. Importantly, CT and a month of follow-up were conducted based on clinical guidelines to ensure that stroke cases were not missed, which could have significantly minimized the risk of misclassification bias (68). Besides, blood routine, hsCRP and uric acid were not available for all patients and were usually available in patients at a high risk of stroke. Missing values of these variables were imputed for analysis to enhance the precision of the established model. LASSO is generally considered superior to logistic regression since the predictive model constructed by LASSO is more stable and handles the problem of correlated inputs. However, only a limited number of features can be selected, and model interpretability is low in cases of low dimension. Finally, the number of stroke events associated with each predictor was small and combined with the relatively limited sample size, might restrict the applicability of the model.

## Conclusion

Our dynamic nomogram, which incorporates clinical data, symptoms, and physical examination, has significant value for estimating the probability of stroke in patients presenting to the ED with dizziness. If future validation is conducted, this tool may have huge prospects for helping EPs differentiate stroke from non-stroke dizziness and assist in therapeutic decision-making during clinical practice.

## Data Availability Statement

The raw data supporting the conclusions of this article will be made available by the authors, without undue reservation.

## Ethics Statement

The studies involving human participants were reviewed and approved by the Ethics Committee of Tongji Medical College, Huazhong University of Science and Technology (S227). The patients/participants provided their written informed consent to participate in this study.

## Author Contributions

YB contributed to the concept and design, data analysis and interpretation, and writing manuscript. FC performed data interpretation, writing, and final approval of the manuscript. All authors contributed to the article and approved the submitted version.

## Conflict of Interest

The authors declare that the research was conducted in the absence of any commercial or financial relationships that could be construed as a potential conflict of interest.

## Publisher's Note

All claims expressed in this article are solely those of the authors and do not necessarily represent those of their affiliated organizations, or those of the publisher, the editors and the reviewers. Any product that may be evaluated in this article, or claim that may be made by its manufacturer, is not guaranteed or endorsed by the publisher.

## Abbreviations

CI: confidence interval
ED: emergency department
EP: emergency physician
ROC: receiver operating characteristic curve
AUC: area under the receiver operating characteristics curve
AIS: acute ischemic stroke
NIHSS: National Institutes of Health Stroke Scale
MRI: magnetic resonance imaging
CT: computed tomography
mRS: modified Rankin Scale
DWI: diffusion-weighted imaging
LASSO: the least absolute shrinkage and selection operator
GEN: gaze-evoked nystagmus.
