# Supplementary material for: A Dynamic Nomogram to Predict the Risk of Stroke in Emergency Department Patients With Acute Dizziness
Source: Front Neurol. 2022 Feb 18;13:839042. doi: 10.3389/fneur.2022.839042 (PMC8896851; doi:10.3389/fneur.2022.839042)
Supplement: Supplementary file 1 [file Supplementary_Material.docx]

**Supplementary materials：**

**
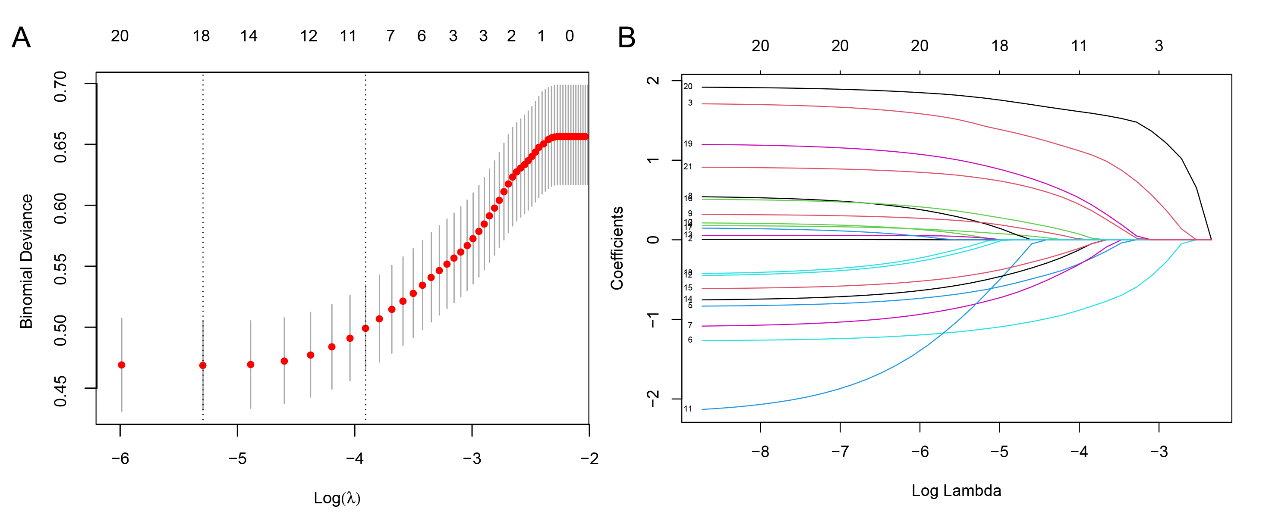
**

**Figure S1**. Selection of predictors using the LASSO logistic regression model. **(A)** Identification of lambda (λ), or the optimal penalization coefficient in the Lasso model using the 1 standard error of the minimum criterion and 10-fold cross validation. **(B)** The plot of each candidate predictors’ coefficient versus log(lambda) by adjusting the parameter lambda value


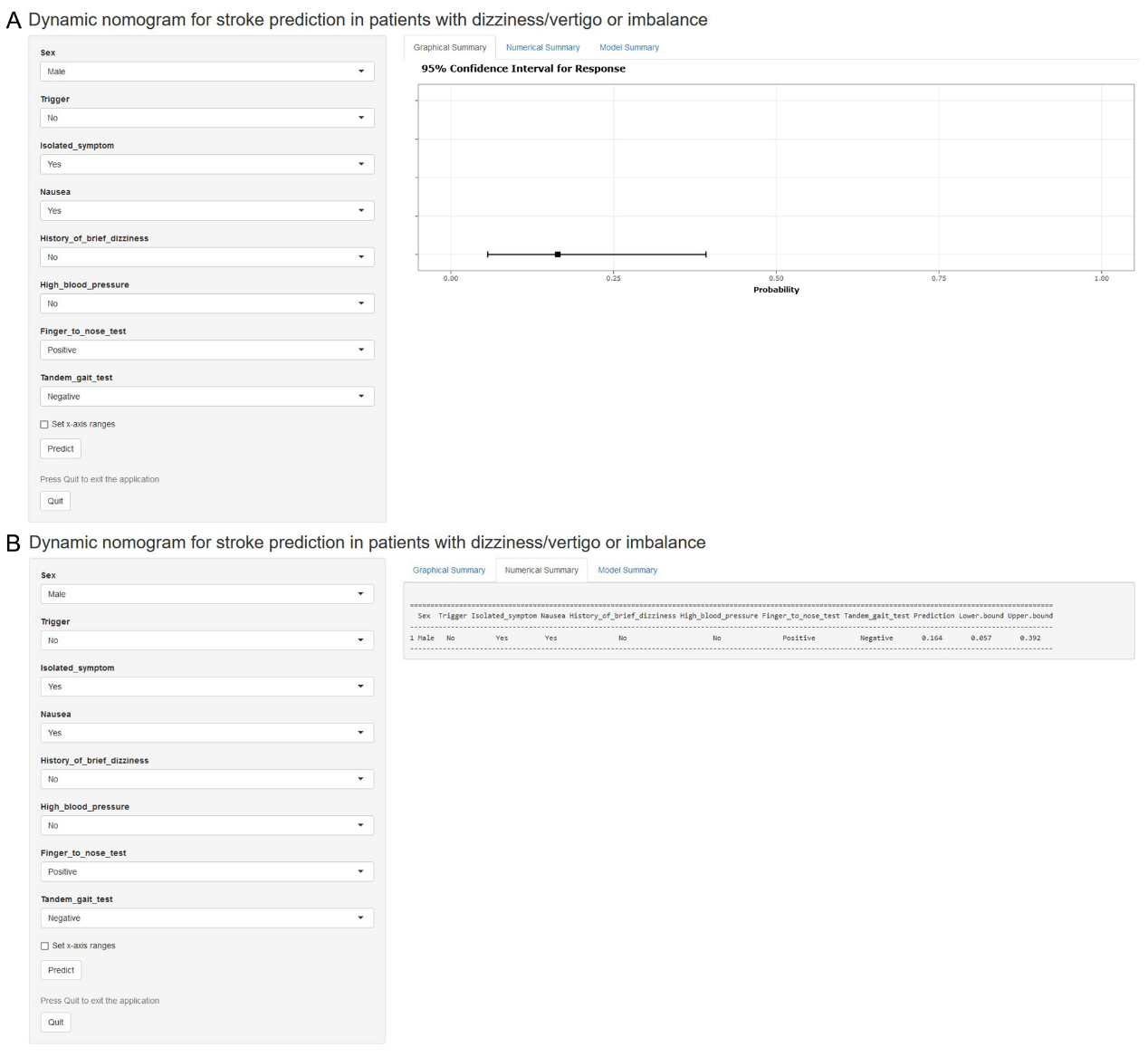


**Figure S2**. The dynamic nomogram of stroke prediction. For example, if a male, isolated, dizziness patient with nausea was admitted at the emergency department; he had no history of brief dizziness within 3 months, and physical examination showed high blood pressure, positive finger to nose test, and negative tandem gait test. The probability of stroke was determined to be 0.164 [95% confidence interval (CI): 0.057–0.392] **(A, B)**.


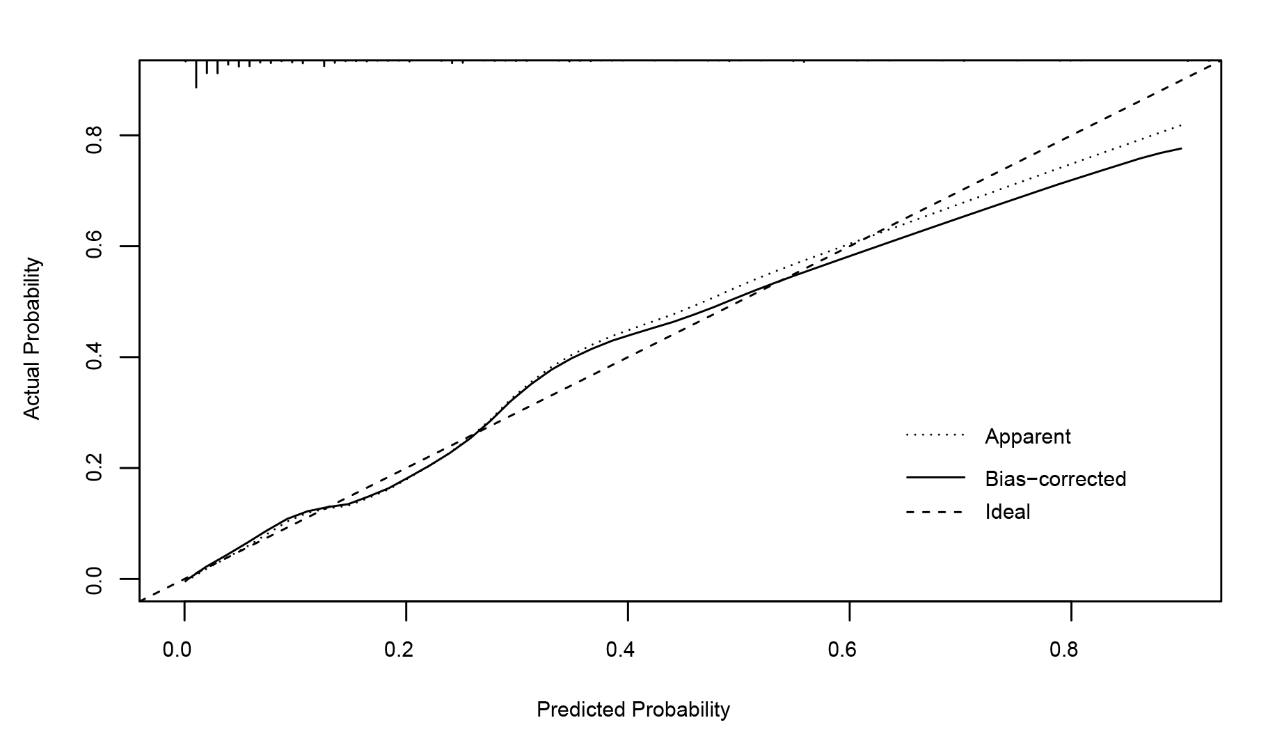


**Figure S3**. Calibration curve of the nomogram model for the prediction of stroke. The x-axis is the predicted probability of stroke, and the y-axis is the actual stroke occurrence rate. The diagonal dashed line represents the ideal model prediction performance; the solid line represents the prediction performance of the nomogram model in this study. The closer it is to the diagonal dashed line, the better the prediction performance

| **Table S1**. Results of Multivariate Logistic Regression Analysis | | | | |
| --- | --- | --- | --- | --- |
| Intercept and variable | β | OR | 95%CI | P-value |
| Intercept | -2.9527 | 0.052 | 0.019-0.135 | < 0.001 |
| Male | 1.7477 | 5.741 | 3.061-11.485 | < 0.001 |
| Trigger | -0.8543 | 0.426 | 0.231-0.761 | 0.005 |
| Isolated symptom | -1.3278 | 0.265 | 0.149-0.471 | < 0.001 |
| Nausea | -1.0642 | 0.345 | 0.188-0.625 | < 0.001 |
| History of brief dizziness | -0.7783 | 0.459 | 0.240-0.843 | 0.015 |
| High blood pressure | 1.3763 | 3.96 | 1.996-8.513 | < 0.001 |
| Finger to nose test | 1.9713 | 7.18 | 3.118-16.770 | < 0.001 |
| Tandem gait test | 1.1077 | 3.028 | 1.678-5.571 | < 0.001 |
|  |  |  |  |  |
